# Systemic immune-inflammation index is associated with coronary heart disease: a cross-sectional study of NHANES 2009–2018

**DOI:** 10.3389/fcvm.2023.1199433

**Published:** 2023-07-07

**Authors:** Jiwen Ma, Ka Li

**Affiliations:** West China School of Nursing, West China Hospital, Sichuan University, Chengdu, China

**Keywords:** coronary heart disease, cross-sectional study, predictive biomarker, systemic immune-inflammation index, association analysis

## Abstract

**Background:**

Inflammation has been linked to the development of coronary heart disease (CHD). The systemic immune inflammation index (SII) is a useful biomarker of systemic inflammation. Our study aimed to explore the correlation between SII and CHD.

**Methods:**

We conducted a multivariate logistic regression analysis, smoothing curve fitting, and segmented model comparison on 15,905 participants with a CHD prevalence of 3.31% and a mean age of 46.97 years.

**Results:**

Adjusting for gender, age, and race, we found a negative association between SII and CHD [odds ratio (OR) 0.66; 95% confidence interval (CI) 0.48, 0.90]. There was an inverse trend where increasing SII was associated with decreasing odds of CHD (*p* for trend = 0.0017). After further adjustment, the association was strengthened, with a similar trend (*p* for trend = 0.0639). Smoothing curve fitting demonstrated a gender-specific association between SII and CHD.

**Conclusions:**

Our findings suggest that higher SII values may be associated with a higher incidence of CHD, which varies by gender. SII may be a cost-effective and convenient method to detect CHD. Further studies are needed to confirm the causality of these findings in a larger prospective cohort.

## 1. Introduction

Coronary artery disease is a common heart disease and a leading cause of morbidity and mortality worldwide (1, 2). It causes approximately 1.78 million European and 360,000 American deaths each year (3). Coronary heart disease is a major cause of morbidity in developed countries and a major driver of healthcare-related costs (4). Coronary heart disease (CHD) accounts for 27% of Europe's total cost of cardiovascular disease (CVD). Patients with CHD often suffer from or even die from cardiovascular events, such as heart failure, stroke, myocardial infarction, and cerebral thrombosis (2). And in the United States, one in six deaths is due to coronary heart disease (4).

The pathological process of coronary heart disease includes atherosclerosis (the main cause) and spasm of the coronary arteries. Lipid metabolic disease, Endothelium damage, inflammation, and immune dysfunction can promote the occurrence and development of coronary atherosclerosis, which leads to CHD (3). Vulnerable plaque is easy to rupture, instability, and easy to forms thrombus plaque. Inflammation plays an important role in vulnerable plaque formation and plaque rupture, which can trigger the formation of blood clots and eventually lead to myocardial infarction (5).

The systemic immune-inflammation index (SII), a novel comprehensive inflammatory biomarker based on neutrophil, lymphocyte, and platelet counts (in 109 cells/L) (6), was created in 2014 by Hu et al., to reflect local immune responses and systemic inflammation (7). Inflammation plays a key role in the occurrence and development of coronary heart disease (CHD) (5). Studies have shown that SII is associated with a poor prognosis of coronary heart disease (CHD) (8). SII can also predict the survival of patients with a variety of tumor prognoses, screening and identification of high-risk patients have applied value (9–11). SII, as an off-the-shelf prognostic biomarker, can help clinicians rapidly identify high-risk patients and make better medical decisions in clinical practice. We hypothesized that SII is a predictor of coronary heart disease risk (12). We conducted the current study to investigate the association between SII and coronary heart disease.

## 2. Materials and methods

### 2.1. Data sources

Data were obtained from NHANES, a national population-based cross-sectional survey evaluated by the National Center for Health Statistics (NCHS) to collect information on potential health risk factors and nutritional status of non-institutionalized civilians in the United States. It uses a complex multistage probabilistic design to recruit a representative sample of the entire U.S. population (13–16). The protocol for the NHANES study was approved by the NCHS Research Ethics Review Committee and written informed consent was obtained from all survey participants. Participants completed a household interview to provide demographic, socioeconomic, and health-related information. Participants underwent a physical examination and laboratory tests at a mobile examination center (MEC) to assess their medical and physiological status. All detailed NHANES study designs and data are publicly available at https://www.cdc.gov/nchs/nhanes/. This study followed the reporting guidelines of the Strengthening the Reporting of Observational Studies in Epidemiology (STROBE) cross-sectional study.

### 2.2. Study population

**Figure 1:**
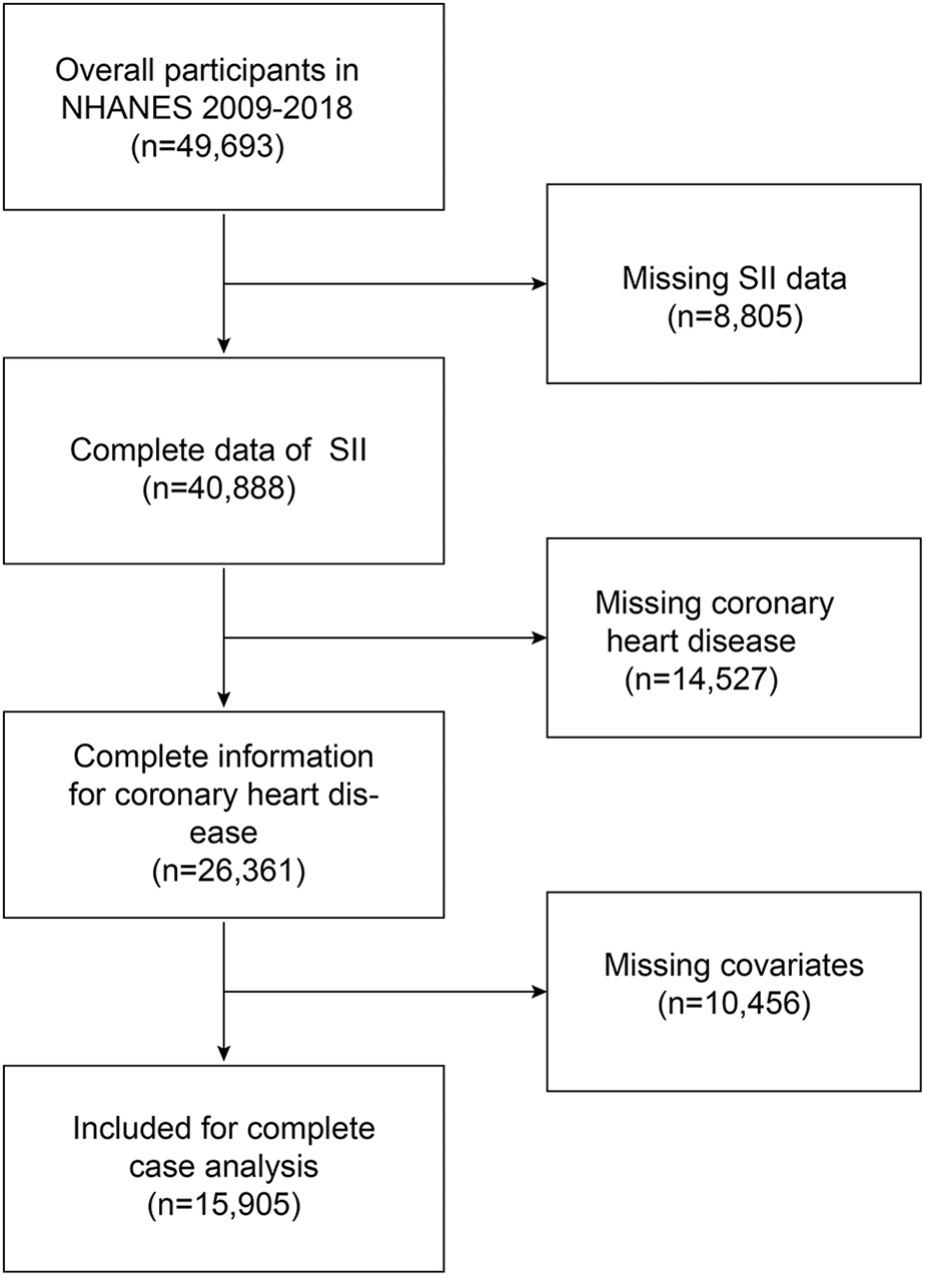
Overview of participants screening. NHANES, National Health, and Nutrition Examination Survey; SII, systemic immune-inflammation index. Definition and grouping of systemic immune-inflammation index (SII) and coronary artery disease (CHD).

Our study combined five NHANES cycles (2009–2010, 2011–2012, 2013–2014, 2015–2016, and 2017–2018) and included the complete set of variables, recruiting a total of 49,693 participants, with exclusion criteria for subjects in our analysis being (1) missing data for SII (2), missing data for coronary artery disease, and (3) missing covariate data. A total of 15,905 participants were initially recruited; after excluding missing SII (*n* = 8,805), missing coronary heart disease (*n* = 14,527), and missing covariates (*n* = 10,456), 15,905 eligible participants ≥20 years of age were included in our final analysis (Figure 1).

### 2.3. Definition of systemic immune-inflammation index

The definition of SII is as follows: SII = P × N/L, where P, N, and L are peripheral platelet, neutrophil, and lymphocyte counts, respectively (7). In our analysis, SII was designed as an exposure variable.

### 2.4. Definition of coronary heart disease

In the health questionnaire, participants were asked “Ever told you had coronary heart disease” (3, 17), and if the answer was “yes”, the individual was considered to have coronary heart disease.

According to previous studies, participants were equally divided into four groups according to the SII distribution: low SII (Q1, <317.5), low-moderate SII (Q2, 317.5–445.7), intermediate SII (Q3, 445.7–625.1) and high SII (Q4, >625.7).

### 2.5. Covariates

Demographic parameters included gender (male/female), age (years), race (Mexican American/Other Hispanic/Non-Hispanic White/Non-Hispanic Black/Other race), marital status (with/without a partner), an education level (above/below high school), smoking (smoker/non-smoker), alcohol consumption (less/more than 15 drinks in the past year), BMI (normal/overweight/obese), household income to poverty ratio (PIR) (18, 19), total cholesterol, triglycerides, total protein, albumin, globulin, serum glucose, serum calcium, and leukocyte platelet, neutrophil, lymphocyte, and monocyte counts were included in the biochemical profile. The NLR is the ratio of neutrophils to lymphocytes. Health risk factors including angina and hypertension were recorded as “yes/no” (20–22).

### 2.6. Statistical analysis

All statistical analyses were conducted according to Centers for Disease Control and Prevention (CDC) guidelines, and an appropriate NHANES sampling all statistical analyses were conducted according to Centers for Disease Control and Prevention (CDC) guidelines and an appropriate NHANES sampling weight was applied, and accounted for complex multistage cluster survey design in the analysis.

Means ± standard errors (SE) were used to describe the distribution of sample means for continuous variables, and frequencies (percentages) were used for categorical variables. To explore the relationship between SII and coronary heart disease (CHD), multivariate logistic regression analysis was performed in three different models considering NHANES complex sampling design (sampling weight). In Model 1, the covariates were not adjusted. Model 2 was minimally adjusted for gender, age, and race. Model 3 was adjusted for age, gender, race, marital status, education level, smoking, BMI, household income-to-poverty ratio (PIR), total cholesterol, triglyceride, total protein, albumin, globulin, serum glucose, serum calcium, and white blood cell platelet, neutrophil, lymphocyte, and monocyte count. In addition, we performed subgroup analyses. All continuous covariates were converted to categorical variables and interaction effects were assessed by likelihood ratio tests. At the same time, we also used smoothed curve fitting to detect non-linearity between SII and coronary artery disease, segmented model comparison to find inflection points, and further explore their threshold effects. Forest plots show each model's hazard ratios and 95% confidence intervals of covariates.

All analyses were performed utilizing R software version 4.1 (http://www.R-project.org; The R Foundation) and EmpowerStats (http://www.empowerstats.com, X&Y Solutions, Inc.). *p* < 0.05 (two-sided) was set for a significant difference.

## 3. Results

### 3.1. Baseline characteristics of the population stratified by SII

This study analyzed data from 15,905 participants with a mean age of 46.97 years ± 17.04 years and a CHD prevalence of 3.31%. The study population was categorized into Qs (Q1–Q4) based on their SII levels. The Qs were found to have significant differences in age, gender, race, marital status, BMI, smoking, total protein, albumin, globulin, triglycerides, and glucose levels (*p* < 0.05), while there were no significant differences in education level, the ratio of family income to poverty, drinking, total calcium, and cholesterol levels among the Qs (Table 1).

**Table 1:** Characteristics of participants in NHANES 2009–2018 (*n* = 15,905).

Characteristics	Q1	Q2	Q3	Q4	*p*-value
	*N* = 3,976	*N* = 3,976	*N* = 3,976	*N* = 3,977	
Age, (years)	47.02 ± 16.88	46.22 ± 16.70	46.70 ± 16.98	48.04 ± 17.54	<0.001
Gender, %					<0.001
Male	2,320 (58.35)	2,215 (55.71)	2,031 (51.08)	1,873 (47.10)	
Female	1,656 (41.65)	1,761 (44.29)	1,945 (48.92)	2,104 (52.90)	
Race, %					<0.001
Mexican American	517 (13.00)	578 (14.54)	610 (15.34)	547 (13.75)	
Other Hispanic	361 (9.08)	425 (10.69)	420 (10.56)	386 (9.71)	
Non-Hispanic white	1,292 (32.49)	1,730 (43.51)	1,850 (46.53)	2,020 (50.79)	
Non-Hispanic black	1,262 (31.74)	751 (18.89)	630 (15.85)	592 (14.89)	
Other race	544 (13.68)	492 (12.37)	466 (11.72)	432 (10.86)	
Education, %					0.514
Below high school	717 (18.03)	744 (18.71)	739 (18.59)	698 (17.55)	
Above high school	3,259 (81.97)	3,232 (81.29)	3,237 (81.41)	3,279 (82.45)	
Marital status, %					0.017
Without partner	1,607 (40.42)	1,531 (38.51)	1,630 (41.00)	1,666 (41.89)	
With partner	2,369 (59.58)	2,445 (61.49)	2,346 (59.00)	2,311 (58.11)	
BMI, %					<0.001
Normal	1,317 (33.12)	1,191 (29.95)	1,099 (27.64)	1,078 (27.11)	
Overweight	1,360 (34.21)	1,355 (34.08)	1,304 (32.80)	1,208 (30.37)	
Obese	1,299 (32.67)	1,430 (35.97)	1,573 (39.56)	1,691 (42.52)	
The ratio of family income to poverty	2.69 ± 1.65	2.71 ± 1.67	2.71 ± 1.67	2.62 ± 1.64	0.066
Smoking, %					<0.001
Non-smoker	2,175 (54.70)	2,122 (53.37)	2,133 (53.65)	1,922 (48.33)	
Smoker	1,801 (45.30)	1,854 (46.63)	1,843 (46.35)	2,055 (51.67)	
Drinking (past 12 mos), %					0.192
<15	3,953 (99.42)	3,938 (99.04)	3,949 (99.32)	3,942 (99.12)	
≥15	23 (0.58)	38 (0.96)	27 (0.68)	35 (0.88)	
Total protein (g/dl)	7.17 ± 0.46	7.14 ± 0.44	7.13 ± 0.44	7.13 ± 0.48	<0.001
Albumin (g/dl)	4.27 ± 0.34	4.29 ± 0.32	4.26 ± 0.34	4.19 ± 0.37	<0.001
Globulin (g/dl)	2.90 ± 0.47	2.85 ± 0.43	2.87 ± 0.43	2.94 ± 0.45	<0.001
Total calcium (mg/dl)	9.38 ± 0.34	9.39 ± 0.36	9.38 ± 0.35	9.38 ± 0.37	0.847
Cholesterol (mg/dl)	191.63 ± 41.50	192.88 ± 42.19	192.87 ± 40.92	192.79 ± 41.61	0.345
Triglycerides (mg/dl)	144.21 ± 129.72	152.38 ± 129.98	154.79 ± 119.71	149.82 ± 115.62	<0.001
Glucose, serum (mg/dl)	99.11 ± 33.90	100.11 ± 34.17	101.05 ± 35.91	103.32 ± 38.79	<0.001
Platelet count (1,000 cells/µl)	202.52 ± 48.01	228.11 ± 46.74	246.91 ± 51.64	277.41 ± 68.67	<0.001
Segmented neutrophils num (1,000 cell/µl)	2.87 ± 1.07	3.75 ± 0.99	4.48 ± 1.18	5.88 ± 2.07	<0.001
Lymphocyte number (1,000 cells/µl)	2.60 ± 6.13	2.23 ± 0.66	2.09 ± 0.65	1.88 ± 0.65	<0.001
White blood cell count (1,000 cells/µl)	6.22 ± 6.87	6.76 ± 1.67	7.40 ± 1.85	8.63 ± 2.58	<0.001
Monocyte number (1,000 cells/µl)	0.52 ± 0.21	0.54 ± 0.17	0.57 ± 0.19	0.61 ± 0.22	<0.001
NLR	1.23 ± 0.39	1.74 ± 0.38	2.23 ± 0.51	3.38 ± 1.54	<0.001
High blood pressure, %					<0.001
Yes	1,283 (32.27)	1,179 (29.65)	1,295 (32.57)	1,463 (36.79)	
No	2,693 (67.73)	2,797 (70.35)	2,681 (67.43)	2,514 (63.21)	
Angina/Angina pectoris, %					0.669
Yes	83 (2.09)	75 (1.89)	75 (1.89)	88 (2.21)	
No	3,893 (97.91)	3,901 (98.11)	3,901 (98.11)	3,889 (97.79)	
Coronary heart disease, %					0.162
Yes	149 (3.75)	113 (2.84)	134 (3.37)	131 (3.29)	
No	3,827 (96.25)	3,863 (97.16)	3,842 (96.63)	3,846 (96.71)	

Data were *n* (%) or mean ± SD. BMI, body mass index, BMI was categorized as normal (<25 kg/m^2^), overweight (25–29.9 kg/m^2^), and obese (≥30 kg/m^2^); SII, systemic immune-inflammation index; the NLR was calculated as the ratio of the neutrophils and lymphocyte; SII quartile: Q1:<317.5; Q2: 317.5–445.7; Q3: 445.7–625.1; Q4: >625.7.

### 3.2. Relationship between SII and coronary heart disease

To better quantify the effect size, we multiplied the SII by 100 (SII percentile) (Table 2). At first, we found no significant association between the SII percentile and CHD (OR 0.98, 95% CI: 0.73, 1.33, *p* = 0.9169) in the non-adjusted model. However, after adjusting for gender, age, and race in the adjusted model I, the association became significant (OR 0.66, 95% CI: 0.48, 0.90, *p* = 0.0086), indicating that higher SII percentiles were associated with lower odds of CHD, and this association showed a trend of decreasing odds of CHD with increasing SII percentile (*p* for trend = 0.0017). Further adjustment for additional variables in adjusted model II strengthened the association, with an OR of 0.74 (95% CI: 0.40, 1.36) and a trend showing decreasing odds of CHD with increasing SII percentile (*p* for trend = 0.0639). The OR for SII Qs also showed a significant trend in Adjusted models I and II, with Q2 showing a lower OR than Q1, and Q4 showing a significantly lower OR than Q1. However, the OR for Q3 was not significantly different from that for Q1. These findings suggest that higher SII values may be associated with a higher incidence of CHD (see Table 2).

**Table 2:** Associations between the systemic immune-inflammation index and coronary heart disease.

Variable	Non-adjusted	Adjust I	Adjust II
	OR (95% CI)	OR (95% CI)	OR (95% CI)
	*p*-value	*p*-value	*p*-value
SII percentile	0.98 (0.73, 1.33)	0.66 (0.48, 0.90)	0.74 (0.40, 1.36)
	0.9169	0.0086	0.3342
SII (Quartile)			
Q1	1.0 (Ref)	1.0 (Ref)	1.0 (Ref)
Q2	0.75 (0.59, 0.96)	0.72 (0.55, 0.93)	0.73 (0.53, 1.00)
	0.0242	0.0136	0.0526
Q3	0.90 (0.71, 1.14)	0.79 (0.62, 1.02)	0.83 (0.59, 1.16)
	0.3641	0.0719	0.2760
Q4	0.87 (0.69, 1.11)	0.63 (0.48, 0.81)	0.61 (0.39, 0.95)
	0.2728	0.0003	0.0292
*p* for trend	0.6215	0.0017	0.0639

The non-adjusted model adjusts for: none. Adjust I model to adjust for: gender (male-female), age (year), and race (Mexican American, other Hispanic, non-Hispanic White, non-Hispanic black, and other race). Adjust II models adjust for Gender (male-female), age (year), race (Mexican American, other Hispanic, non-Hispanic White, non-Hispanic black, and other race), marital status (with or without a partner), educational level (above high school/below high school), smoking (smoker/non-smoker), drinking (drinking less than or more than 15 drinks in past 12 mos), BMI (normal/overweight/obesity), household income-to-poverty ratio (PIR), cholesterol, triglyceride, total protein, albumin, globulin, serum glucose, serum calcium and white blood cell platelet, neutrophil, lymphocyte, monocyte count, Angina and hypertension (Yes/No). SII grouping standard: Q1: <317.5; Q2: 317.5–445.7; Q3: 445.7–625.1; Q4: >625.7.

### 3.3. Subgroup analysis

**Figure 2:**
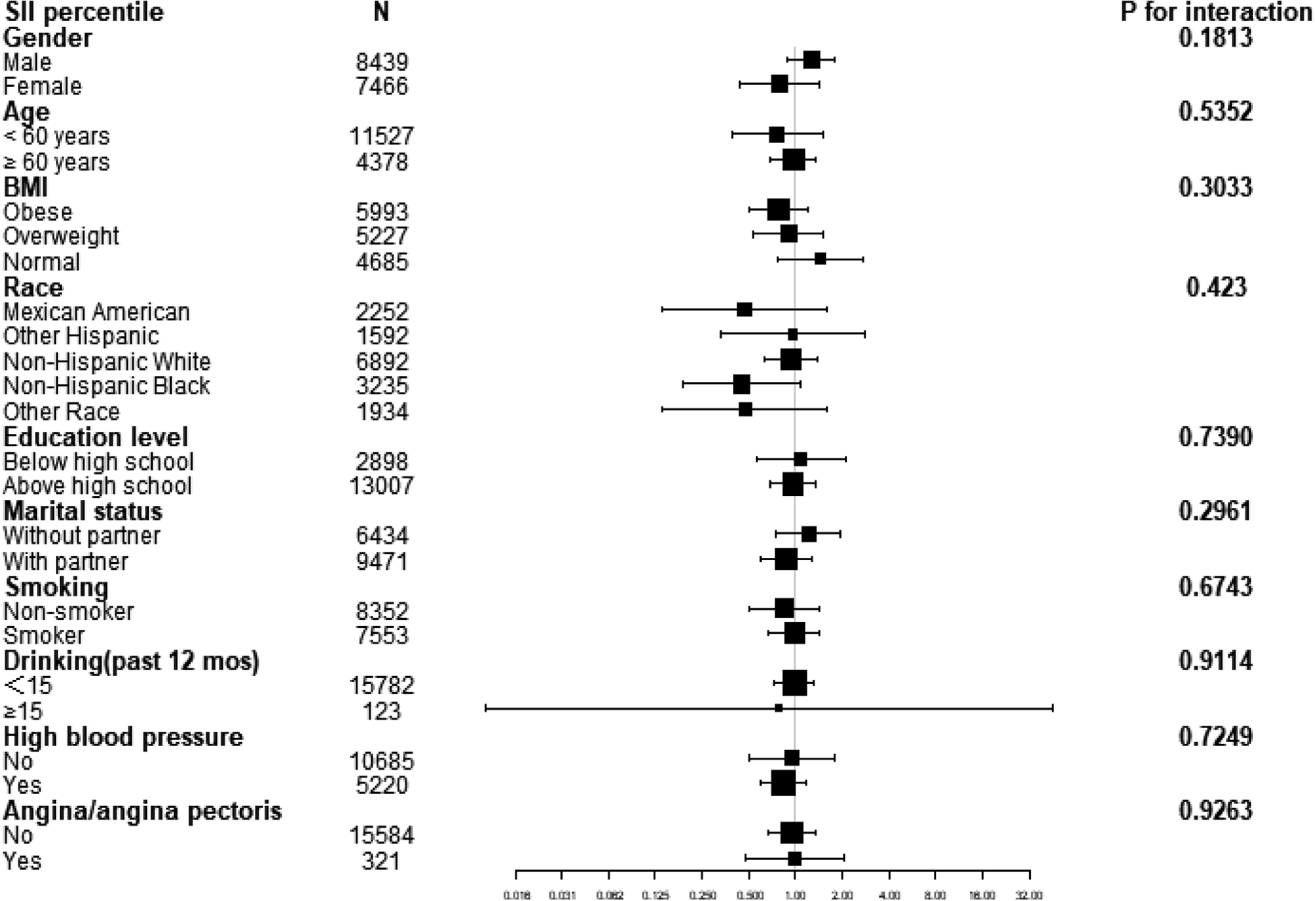
Forest diagram of interaction effects.

A subgroup analysis was conducted to investigate the association between the SII percentile and CHD across various demographic and clinical characteristics, as shown in Figure 2. The subgroups included gender, age, BMI, race, education level, marital status, smoking, drinking, high blood pressure, and angina/angina pectoris. The results of the subgroup analysis indicated no statistically significant associations between the SII percentile and CHD in any of the subgroups, with all *p*-values >0.05. However, it is worth noting that there is a suggestion of a positive association between the SII percentile and high blood pressure in the “yes” subgroup, as the odds ratio (OR) is less than 1 and the *p*-value is close to 0.05 (Table 3).

**Table 3:** Subgroup analysis of SII percentile.

SII percentile	*N*	OR (95% CI)	*p*-value	*p* for interaction
Gender				0.1813
Male	8,439	1.27 (0.89,1.80)	0.1916	
Female	7,466	0.80 (0.44,1.42)	0.4401	
Age				0.5352
<60 years	11,527	0.76 (0.39,1.49)	0.4263	
≥60 years	4,378	0.97 (0.69,1.36)	0.8428	
BMI				0.3033
Obese	5,993	0.78 (0.50,1.22)	0.2828	
Overweight	5,227	0.90 (0.53,1.52)	0.6852	
Normal	4,685	1.44 (0.76,2.75)	0.2627	
Race				0.423
Mexican American	2,252	0.47 (0.14,1.61)	0.2284	
Other Hispanic	1,592	0.96 (0.33,2.82)	0.9444	
Non-Hispanic white	6,892	0.93 (0.63,1.37)	0.7114	
Non-Hispanic black	3,235	0.45 (0.19,1.07)	0.0703	
Other race	1,934	0.48 (0.14,1.58)	0.2244	
Education level				0.7390
Below high school	2,898	1.09 (0.56,2.10)	0.8021	
Above high school	13,007	0.96 (0.68,1.35)	0.8096	
Marital status				0.2961
Without partner	6,434	1.21 (0.74,1.97)	0.4406	
With partner	9,471	0.87 (0.59,1.28)	0.4786	
Smoking				0.6743
Non-smoker	8,352	0.85 (0.51,1.42)	0.5425	
Smoker	7,553	0.98 (0.67,1.42)	0.9020	
Drinking (past 12 mos)				0.9114
<15	15,782	0.99 (0.73,1.33)	0.9276	
≥15	123	0.78 (0.01,45.87)	0.9058	
High blood pressure				0.7249
No	10,685	0.95 (0.50,1.78)	0.8677	
Yes	5,220	0.83 (0.59,1.17)	0.2935	
Angina/angina pectoris				0.9263
No	15,584	0.95 (0.67,1.35)	0.7605	
Yes	321	0.98 (0.48,2.03)	0.9639	

The results of the subgroup analysis were adjusted for all covariates except the effect modifier. 95% CI, 95% confidence interval; OR, odds ratio; BMI was categorized as normal (<25 kg/m^2^), overweight (25–29.9 kg/m^2^), and obese (≥30 kg/m^2^); SII, systemic immune-inflammation index.

**Figure 3:**
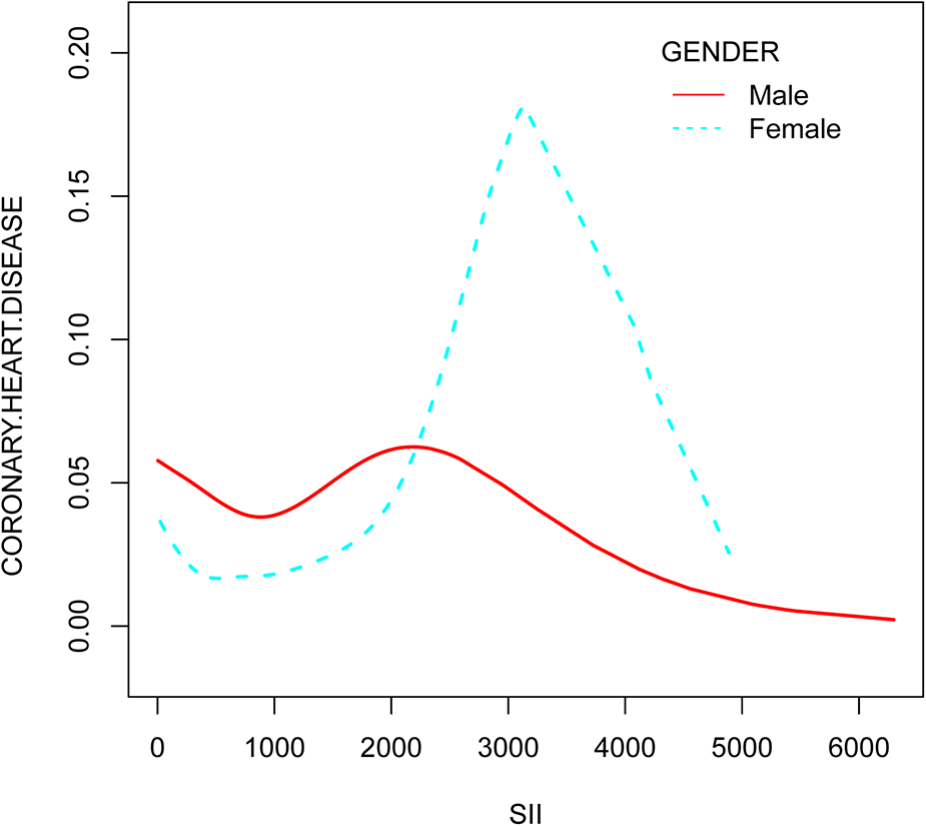
The relationship between systemic immune-inflammation index and coronary heart disease risk stratified by gender.

The smooth curve fitting analysis showed an inverted U-shaped relationship between SII and CHD risk in both genders, with a significant effect above the fold point (in Figure 3).

## 4. Discussion

This study analyzed data from the NHANES database for 2009–2018. In our cross-sectional study of 15,905 participants, a negative association between SII and coronary heart disease was observed across quartiles of SII, and smooth curve fitting shows the same result. Subgroup analyses and interaction tests showed that the association was similar across population settings.

In recent years, blood cell-based inflammatory parameters, including white blood cells, neutrophil, and neutrophil to lymphocyte ratios, have received increasing attention because they can predict some diseases, such as blunted rest-activity rhythm, psychiatry problems, and cardiovascular anomalies (6). Many studies have demonstrated that SII has a remarkable predictive ability (3, 6–9, 12, 22–29). Systemic immune inflammatory index (SII) was initially defined as the prognosis of cancer, intracerebral hemorrhage, and coronary artery stenosis (23). However, the relationship between SII itself and coronary heart disease has not been studied. SII measurements are based on standard laboratory methods of peripheral neutrophils, lymphocytes, and platelets in clinical practice. Simultaneously, SII was a widely available method with a non-intrusive methodology, simple accessibility, and low cost. The potential for therapeutic use is indeed positive (25). Arteriosclerosis is the basic mechanism of coronary heart disease. Abnormal apoptosis of Endothelium cells (VEC), macrophages, or vacs is a common feature of arteriosclerosis, leading to the formation or destabilization of arteriosclerosis plaques (1). Studies have shown that SII is associated with mortality of the cardiovascular system in patients (10, 11). Inflammation plays an important role in the formation and development of arteriosclerosis, and studies have found that they can serve as risk stratification markers and predict adverse events (30). Inflammation-endothelial dysfunction interaction triggers and promotes arteriosclerosis (5). Elevated inflammatory markers increase the risk of cardiovascular disease (CVD), but the underlying mechanisms and pathways remain to be elucidated (28). SII can reflect local immune response and systemic inflammatory response and is an important inflammatory marker. SII consists of three types of blood cells: platelets, neutrophils, and lymphocytes (30). Studies have shown that increased neutrophils can promote oxidative damage to blood vessel walls, while decreased lymphocytes can exacerbate oxidative and inflammatory damage (20, 30, 31). The neutrophil interacts with platelets to influence important biological processes related to arteriosclerosis, thrombosis, and ischemic attack. Following apoptosis of lymphocytes, the lipid core of atherosclerotic plaques ruptures and produces a thrombus (27).

Considering the Neyman bias (32), inherent in cross-sectional studies, our ability to ascertain the occurrence of a disease in individuals beyond the survey's time point is limited. We can only estimate disease prevalence based on the survey data, without definitive knowledge of past or future disease status. This constraint applies to our study as well.

Our study utilized NHANES data, benefiting from its reliable and well-designed cross-sectional framework. Notably, we employed a substantial sample size and conducted subgroup analyses to elucidate the distribution of SII in both coronary and non-coronary populations. Furthermore, our analyses included adjustments for relevant covariates, allowing us to explore potential confounding factors linked to coronary heart disease, encompassing sociodemographic and lifestyle variables. We employed various forms of independent variables, such as continuous and categorical variables, constructing robust multiple regression models to yield more reliable findings. Moreover, we applied smooth curve fitting to visually portray the interrelationships between variables in a more accessible manner. Nevertheless, it is imperative to acknowledge the limitations of our study. The cross-sectional design precludes establishing causal relationships, necessitating prospective investigations for causal determination. Additionally, the reliance on self-reported questionnaire data for coronary heart disease status and covariates introduces potential subjective biases. Furthermore, the measurement of immune cell counts was based on a single blood test, while a continuous monitoring approach would provide more reliable data considering the lifespan of blood cells. Notably, we should remain mindful of potential unobserved confounding factors, and further exploration of clinical conditions, such as diabetes, is warranted. Indeed, our study offers valuable insights that contribute to the current body of knowledge. Nonetheless, it is essential to carefully consider the limitations we have discussed when interpreting our findings. These limitations are crucial in providing a comprehensive understanding of the context and implications of our research.

In summary, our study suggests that SII may be a useful marker for predicting CHD risk, particularly in males and females above a certain SII level. However, further research is needed to confirm these findings and to determine the optimal SII level for predicting CHD risk in different genders.

## 5. Conclusion

To sum up, our findings add to the growing body of evidence supporting the clinical utility of SII as a predictive biomarker for disease outcomes. Future prospective studies are needed to establish causality and explore the potential clinical applications of SII in the prevention and treatment of coronary heart disease.

## Data Availability

The datasets presented in this study can be found in online repositories. The names of the repository/repositories and accession number(s) can be found below: https://www.cdc.gov/nchs/nhanes/.
